# How ready are communities to implement actions to improve diets of adolescent girls and women in urban Ghana?

**DOI:** 10.1186/s12889-019-6989-5

**Published:** 2019-05-28

**Authors:** Rebecca Pradeilles, Colette Marr, Amos Laar, Michelle Holdsworth, Francis Zotor, Akua Tandoh, Senam Klomegah, Nathaniel Coleman, Kristin Bash, Mark Green, Paula L. Griffiths

**Affiliations:** 1 Public Health Section, School of Health and Related Research, University of Sheffield, 30 Regent Street, Sheffield, UK; 2 Department of Population, Family & Reproductive Health, School of Public Health, University of Ghana, Accra, Ghana; 3 Department of Family and Community Health, University of Health and Allied Sciences, Ho, Ghana; 4 School of Environmental Sciences, University of Liverpool, Liverpool, UK; 5 School of Sport, Exercise and Health Sciences, Loughborough University, Loughborough, UK

**Keywords:** Community readiness, Interventions, Unhealthy diet, Ghana, Women, Urban

## Abstract

**Background:**

Ghana has reached an advanced stage of nutrition transition, contributing to an increase in nutrition-related non-communicable diseases, particularly amongst urban women. Community involvement is an important factor in the success of efforts to promote healthy eating. The readiness of populations to accept a range of interventions needs to be understood before appropriate interventions can be implemented. Therefore, this study assessed how ready urban communities are to improve diets of women of reproductive age in Ghana.

**Methods:**

Using the Community Readiness Model (CRM), in-depth interviews were conducted with 24 key informants from various sectors in low income communities across two cities in Ghana: Accra and Ho. The CRM consists of 36 open questions addressing five readiness dimensions (community knowledge of efforts, leadership, community climate, knowledge of the issue and resources). Interviews were scored using the CRM protocol with a maximum of 9 points per dimension (from 1 = no awareness to 9 = high level of community ownership). Thematic analysis was undertaken to gain insights of community factors that could affect the implementation of interventions to improve diets.

**Results:**

The mean community readiness scores indicated that both communities were in the “vague awareness stage” (3.35 ± 0.54 (Accra) and 3.94 ± 0.41 (Ho)). CRM scores across the five dimensions ranged from 2.65–4.38/9, ranging from denial/resistance to pre-planning. In both communities, the mean readiness score for ‘knowledge of the issue’ was the highest of all dimensions (4.10 ± 1.61 (Accra); 4.38 ± 1.81 (Ho)), but was still only at the pre-planning phase. The lowest scores were found for community knowledge of efforts (denial/resistance; 2.65 ± 2.49 (Accra)) and resources (vague awareness; 3.35 ± 1.03 (Ho)). The lack of knowledge of the consequences of unhealthy diets, misconceptions of the issue partly from low education, as well as challenges faced from a lack of resources to initiate/sustain programmes explained the low readiness.

**Conclusions:**

Despite recognising that unhealthy diets are a public health issue in these urban Ghanaian communities, it is not seen as a priority. The low community readiness ratings highlight the need to increase awareness of the issue prior to intervening to improve diets.

**Electronic supplementary material:**

The online version of this article (10.1186/s12889-019-6989-5) contains supplementary material, which is available to authorized users.

## Background

Ghana, a lower-middle income country [1], is in the latter stages of the nutrition transition [2, 3]. Although the overall prevalence of overweight and obesity in adults in Ghana is 43%, the prevalence is significantly higher in women than in men (49.7% vs 27.8%), and in urban than rural dwellers (47.8% vs 24.7%) [4]. As women play a key role in society by contributing to the health-care workforce, nutritional adequacy of households, education, and to the care of families and communities [5], promoting healthy eating in women could benefit immediate family members as well as others within the wider community [5]. There is therefore an urgent need for interventions to promote healthier diets to prevent obesity and other forms of malnutrition that reach urban females in Ghana.

Community involvement has been recognised as an important factor in the success and sustainability of efforts to promote healthy eating and prevent obesity [6]. Within a community, the success of an intervention is dependent upon the community’s readiness for change [7]; according to behaviour change theory, strategies should be matched to the stage of readiness [8]. An initial assessment of community readiness should therefore be conducted prior to implementing a community-based intervention.

The CRM provides a theoretical framework for understanding and improving community readiness [7, 9]. Based on the Transtheoretical Model of Behaviour Change for individual readiness [10], the CRM approaches problems that exist in a community by integrating a community’s culture, resources and level of readiness to assess the degree to which a community is prepared to take action on an issue [11]. The CRM provides quantitative scores of readiness level via assessment of in-depth individual interviews conducted with key informants in the community [12]. A qualitative analysis of responses is also recommended to support the quantitative scoring [13].

The CRM has most frequently been used within the U.S.A, for alcohol and drug use [14]. However, the tool has also been used to assess readiness for overweight and obesity prevention in high-income countries (HICs) including the U.S.A [15, 16], Australia [17, 18] and the UK [19] and applied to ethnic minority [20] or disadvantaged communities [21]. Limited research has been conducted in low- and middle-income countries (LMICs) using the CRM. For example, it has been applied to preventing communicable diseases in Bangladesh [22] and Mali [23], and mental health stigma in Vietnam [24]. Only one study (from South Africa) has assessed community readiness for overweight/obesity prevention in Africa [25].

This current study investigated how ready urban communities are to improve the diets of women of reproductive age in Ghana. The specific objectives were: (i) to determine the stage of readiness of communities to address the consumption of unhealthy foods and beverages in adolescent girls and women of reproductive age in two deprived communities at different stages of the nutrition transition in urban Ghana, and (ii) to gain qualitative insights of community factors that could affect the implementation of interventions targeting unhealthy food and beverage consumption.

## Methods

### Study setting

This study was conducted in two Ghanaian cities; the densely populated city of Accra (estimated population of 1.85 million) and Ho (estimated population of 120,349) [26]. These two cities were chosen as they are at different stages of the nutrition transition, as evidenced by differences in the regional prevalence of overweight/obesity; 57.3% in Greater Accra (Accra) and 31.1% in the Volta region (Ho) [27].

Communities within these two cities were selected to represent the lower quintile wealth populations, as the study was most interested in understanding the potential for intervention in the very poorest urban communities in Ghana. A list of all deprived communities in Accra and Ho was produced. In Accra, the selection of a study community was informed by findings of the Accra Poverty Mapping Exercise [28]. The Ga Mashie community in Accra was chosen purposively as it was classified as a high poverty zone. In Ho, the selection of a study community was informed by using the 2009 United Nations Human Settlements Programme urban profiling report [29]. The Ho Central area was purposely chosen for convenience of location and meeting the economic inclusion criteria.

### Data collection

A geographical definition was used to define our communities of interest (Ga Mashie and Ho Central areas in Accra and Ho, as defined by the respective local authorities – Accra Metropolitan Assembly and Ho Municipal Assembly respectively). Key informants with a leadership role within the selected communities were interviewed (*n* = 12 in each community) between February and May 2018 [30]. To ensure a range of views within the communities, key informants were recruited purposively to represent a wide range of sectors (schools, commerce, health, religious institutions, development agencies, traditional authorities and youth clubs). If leaders agreed to participate, they were given a participant information letter and written informed consent was taken on the day of the interview.

**Fig. 1:**
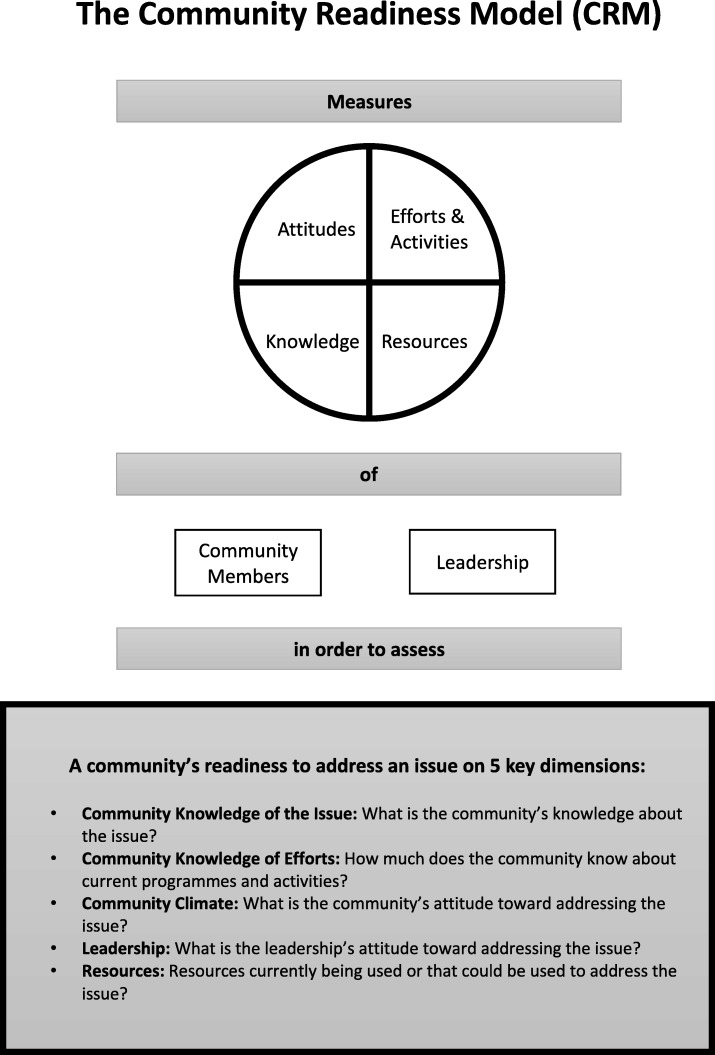
The dimensions of the Community Readiness Model. Source: Adapted from the Tri Ethnic Center Community Readiness Handbook 2nd Edition, page 1 [30]

The CRM tool [30] was administered via individual face to face interviews with key informants. The CRM tool consists of 36 open questions addressing five readiness dimensions: community knowledge of efforts, leadership, community climate, community knowledge of the issue, and resources (Fig. 1). The issue was defined as the “consumption of unhealthy foods and drinks in adolescent girls and women aged 13-49 years”. This referred to the consumption of products such as processed meats; sugar and sweet spreads; cakes and sweets; sodas and sweetened drinks; fried potatoes, yam or plantain; oils; spreading fats; cooking fats; and condiments (such as ketchup, mayonnaise).

Interviews were administered in English. The CRM interview guide was piloted with the local teams and two community key informants. As a result of this, the tool was amended and adapted to the urban Ghanaian context (i.e. re-phrasing of sentences and altering terminology) (Additional file 1). The Likert scales to measure opinions were culturally adapted to facilitate the understanding of the scale system. To keep the interview time relatively short (approximately 30–45 min), the number of questions included in the interview guide was also limited to only those required for scoring readiness as specified by the CRM authors [30].

### Data analysis

**Fig. 2:**
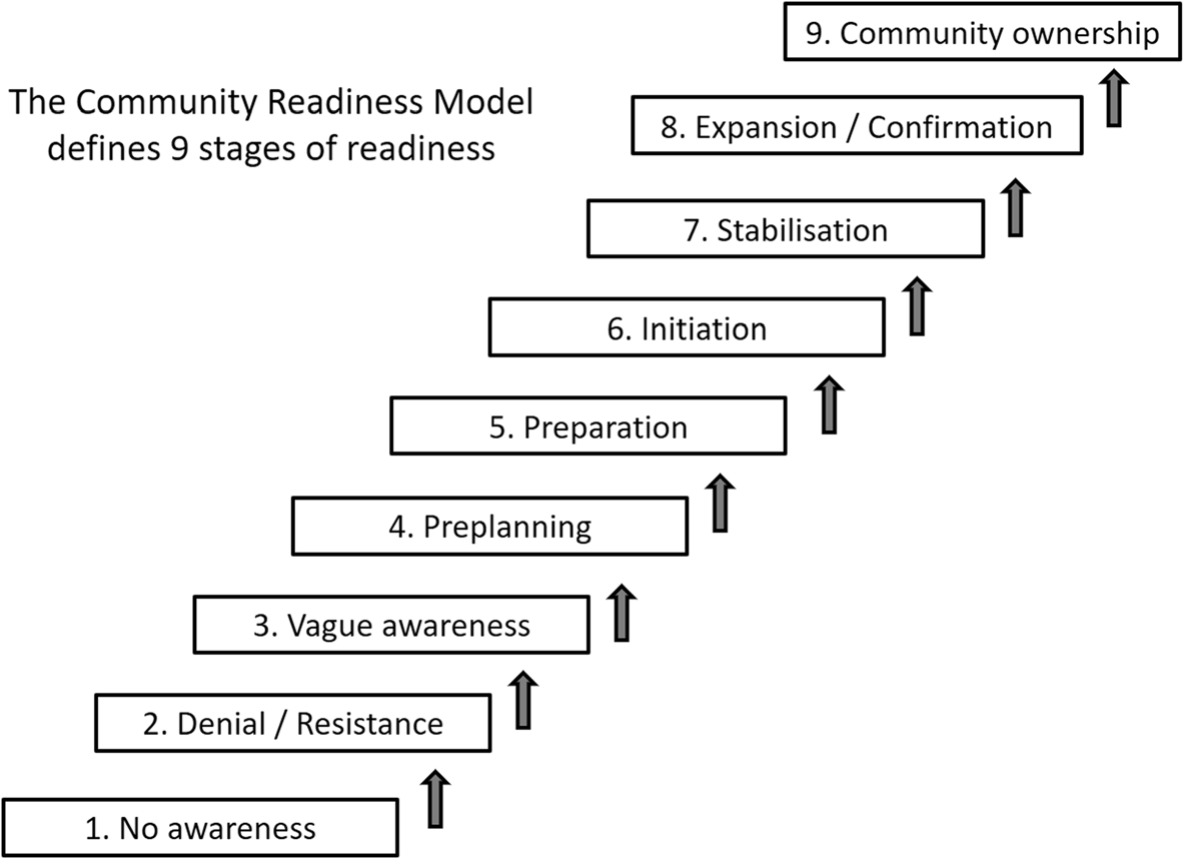
The nine stages of Community Readiness. Source: Adapted from the Tri Ethnic Center Community Readiness Handbook, 2nd Edition, page 6 [30]

Key informant interviews were transcribed verbatim, reviewed for accuracy and scored by two independent scorers (RP/CM or RP/KB) [25, 30]. Each interview was first read through in full to gain a general idea of the content. Scorers then compared each response given by community key informants to the nine anchored rating statements for each dimension and matched it to the most appropriate anchored rating statements [30] (see pages 49–53). After scoring, the average score for each dimension was generated and a score for each key informant was then calculated as the sum of average scores for each dimension divided by the number of dimensions. Finally, an overall stage of community readiness to address consumption of unhealthy foods and beverages in women and adolescent girls was calculated for all key informants. This was obtained by summing the scores for each key informant, divided by the number of key informants included in the study (range 1–9). Scores were generated separately for Ga Mashie and Ho Central to allow comparisons between the two communities. The overall mean score was then compared to the nine stages of community readiness defined in the CRM (Fig. 2) to evaluate the level of readiness, and efforts needed.

Transcripts were also analysed using thematic analysis to allow an in-depth interpretation of the scores obtained [31]. Pattern themes, defined as concepts that occurred frequently across interviews, were identified from the analysis of the coded text. Themes were identified from initial codes if the topic was raised in more than one interview, meaning that an inductive approach was taken. The coders (RP/CM) then agreed on the final codebook to use to code all transcripts (Additional file 2). Coding was undertaken using NVivo version 11. Six of the 24 transcripts (25%) were independently coded by a second coder and final codes were agreed upon discussion.

## Results

### Sample characteristics

Across the two communities, informants interviewed (*n* = 24) had similar roles and were representing similar sectors. In Ga Mashie, the majority of respondents were male (66.7%), whilst in Ho Central there was an equal proportion of male and female respondents. Across both communities, most key informants were above the age of 35 years (Table 1).

**Table 1:** Sample characteristics of community key informants (*n* = 24)

		Ga Mashie (Accra) (*n* = 12)		Ho Central (Ho) (*n* = 12)	
		n	%	n	%
Sex	Male	8	66.7	6	50.0
Age (years)	25–34	2	16.7	2	16.7
	35–44	5	41.7	3	25.0
	55+	5	41.7	7	58.3
Key informant roles/sectors	Non-Governmental Organisations	2	16.7	1	8.3
	Health^a^	2	16.7	2	16.7
	Assembly officers	1	8.3	2	16.7
	Religious leaders^b^	2	16.7	2	16.7
	Traditional leaders	2	16.7	2	16.7
	Youth leaders	1	8.3	1	8.3
	Education^c^	1	8.3	1	8.3
	Commerce	1	8.3	1	8.3

^a^Health workers included nutrition officers, health promotion officers and Food and Drug Administration officers

^b^Religious leaders included Pentecostal, Evangelical Presbyterian, Christian and Islamic leaders

^c^School Health Education Programme coordinators

### Community readiness scores

Mean (standard deviation) community readiness scores were 3.35 ± 0.54 (Ga Mashie) and 3.94 ± 0.41 (Ho Central) which correspond to the “vague awareness stage” (Table 2). This corresponds to the third lowest (of nine) stage of community readiness. This stage is reached when: (i) a few community members have at least heard about local efforts, but know little about them, (ii) the leadership and community members believe that the consumption of unhealthy foods and beverages is a concern in the community but they show no immediate motivation to act, (iii) community members have only vague knowledge about the issue and (iv) there are limited resources within the community to mobilise towards the issue [30].

**Table 2:** Community readiness scores (out of 9) obtained from a sample of key informants in urban Ghana (*n* = 24)

	Knowledge of efforts	Leadership	Community Climate	Knowledge of Issue	Resources
Community mean scores (SD) for Ga Mashie	2.65 (2.49)	3.50 (1.34)	3.42 (0.78)	4.10 (1.61)	3.08 (0.40)
*Overall Readiness Score (SD) for Ga Mashie*	*3.35 (0.54)*	*“Vague Awareness stage”*			
Community mean scores (SD) for Ho Central	3.81 (3.12)	3.85 (1.96)	4.29 (1.32)	4.38 (1.81)	3.35 (1.03)
*Overall Readiness Score (SD) for Ho Central*	*3.94 (0.41)*	*“Vague Awareness stage”*			

SD: standard deviation

In the Ga Mashie community, the mean readiness score for knowledge of the issue was the highest of all the dimensions (4.10 ± 1.61), followed by leadership (3.50 ± 1.34). In Ho Central, the highest scores were displayed for knowledge of the issue (4.38 ± 1.81) and community climate (4.29 ± 1.32). The lowest scores were found for knowledge of efforts (2.65 ± 2.49) and resources (3.35 ± 1.03) in Ga Mashie and Ho Central, respectively.

### Community knowledge of efforts

The scores for community knowledge of efforts were low in both communities. Ga Mashie had the lowest score for this dimension (2.6), which revealed that the Ga Mashie community is in the ‘denial resistance stage’, whereby community members have misconceptions or incorrect knowledge about current efforts. In contrast, scores for Ho Central suggested that they are in a slightly higher readiness stage (3.8) referred to as the ‘vague awareness stage’, reflecting that a few community members have heard about local efforts but know little about them (Table 2).

The low scores across both communities can be attributed to the *many* (Ga Mashie) or *some* (Ho Central) key informants who were unaware of any efforts to address the consumption of unhealthy food and drinks in women of reproductive age (Table 3). In both communities, existing programmes were often only one-off events and conducted in health care facilities, or schools. Consequently, only specific groups were targeted, e.g. pregnant women, patients with non-communicable diseases, or school pupils. The content of such efforts ranged from basic food hygiene to targeting nutrient balance.

**Table 3:** Supporting quotes for ‘community knowledge of efforts’

	Ga Mashie (Accra)	Ho Central (Ho)
Type & content	‘There has been…researchers… to investigate into the dietary patterns among adolescent ladies in this community…those who did the research came back to the community to report back...they did some posters on their findings’...’And then there is another programme that is targeting non-communicable diseases, people that are living with stroke, diabetes, hypertension’ (Male, NGO). ‘We go to the schools, we educate them, we tell them which one *(food and drinks)* is good, which one is not good. Those that can be taken in moderation and those that if there is the will, they could eliminate’ (Male, Education Sector). ‘I have not seen any such efforts. We have NGOs and things coming here but issues of our eating habits and then the rest it doesn’t happen’ (Male, Youth Leader).	‘They *[community health nurses]* talk about health issues, they always talk health issues. I even have a pregnancy class. So once a while, we call them, the pregnant women, we call them and tell them all they should eat’ (Female, Traditional Leader). ‘We don’t know about any such programmes...There is little [*information]...*Normally they are graphic representations...at institutions like the hospitals where you can see one or two things talking about the dangers on eating unsafe foods. But within community you are hardly to get such’ (Male, Health Sector). ‘We now have the SMART schools. Schools that...they are being educated on good nutrition. With the nurse being the facilitator’ (Female, Health Sector). ‘My church… they let the community health nurses to come…and they encourage any age…to come and listen about the kind of food we eat and the rest but aside that, I have not heard anything’ (Female, NGO).
Target group	‘We talk to the pregnant women...we don’t have anything specifically for those who are not pregnant but are in that age *[13–49 yrs]...*we have been educating them as well - The diabetics and the hypertensives’ (Female, Health Sector).	‘In actual fact, I think some of these efforts are done in schools, where the pupils are advised on eating healthy foods. Apart from that…the community health nurses, when they come for weighing also advise the parents on good diets for their children’ (Female, Education Sector).
Facilitator	‘We [*health officers at the polyclinic]* organise and plan everything’ (Female, Health Sector). ‘The Regional Institute of Population Studies [University of Ghana]. They did the study here’ (Male, NGO).	‘Some of these efforts are done in schools... the community health nurses...at times...NESTLE organised a programme’ (Female, Education Sector). ‘I am also aware that the municipal Nutrition Officer has been doing a lot in terms of radio and talks’ (Female, Health Sector). ‘Yes, at the church, programmes like youth programmes, women programmes, they do invite resource persons that are well invested in healthy lifestyle, including nutrition aspect and they do educate them’ (Male, Religious Leader).
Duration and time	‘They probably do something one-off and then that is it and then they may be underlying factors like funding...so they are also not able to implement long-term programmes that will actually lead to behavioural changes’ (Male, NGO).	‘My church... they let the community health nurses to come and…it was a week programme... I was expecting that same kind of programme in my church which never happened again, so it is being assumed that it has been done so that is it’ (Female, NGO).
Community engagement & reach	‘So it is only when they come to the facility that... but in the community to be frank nutrition... I have not gone round...[laughs] I have quite a number of brochures and all that about anaemia in pregnancy, what to eat, what not to eat but I have never sent out to the community to give out. We don’t have anything like that in the community. It is only at the facility’ (Female, Health Sector).	‘Announcements are made to let them know.. at times these information vans go around to inform the people that there will be a talk on nutrition... so all are invited to come’ (Female, Education Sector). ‘Another strength is that the champions, like the model mothers... so now they are also getting the information out to others to replicate the information to more people. So, it is not only the community health nurses, so one of the strengths is that we are able to give the information out to others to who are able to spread it out’ (Female, Health Sector).
Misconceptions about the efforts	‘Well I am being very careful because as I said most of the populace there are semi-literate, so it is just a few that will understand exactly what we are carrying out. The others some may hear it and they have their own interpretation they give to some of these things’ (Male, Education Sector). ‘Normally, these people I will say they think it is just for the elderly’ (Male, NGO).	‘Oh, there is no misconception, they always take it in good faith. So, there isn’t any misconception’ (Male, Youth Leader).
Weaknesses	‘The weaknesses will be the fact that we don’t reach out to a larger group of people and it is most of the time the pregnant women and all that. So that is the number one weakness’ (Female, Health Sector).	‘So, most of the information is not going out as fast as we want it, as it should. The smart school I said, uptake of those programmes in Ho Central is quite difficult, because they tell you they go to the school and international schools and this, so it is difficult to penetrate into the schools, so it is not working as well as it would be working in other districts’ (Female, Health Sector).
Obstacles	‘Influence from relatives…Like we tell them not to eat this and then somebody tells them ooh what is it? you can eat this’. (Female, Health Sector). ‘No they will not oppose but some people will be asking what is in for them. They will ask what they will get from participating in the programme and that will be in terms of financial gains’ (Male, NGO).	‘The obstacles, one is poverty. And also, some are not working, so they lack the means. And some too are single parents’ (Female, Education Sector). ‘Anything you tell them, they don’t partake in activities… even though it is bringing harm to their bodies, they don’t listen. You see them, you talk to them and then the following day, they are more drunk than anything’ (Male, Traditional Leader). ‘Some don’t even have TVs, those who don’t have, they are people in this Ho Municipality that they don’t even have TVs in their homes, so and the person might not even have radio so how will this person get the information’ (Female, NGO).
Strengths	‘So, for those people that are receiving the intervention *[university research]* ... they like the food and the advice on how to prepare the food and what goes into preparing good food’... ‘So, the effort in getting people to participate in such an intervention I think is a strength. They come with vehicles and they are able to move them around so to me the effort from the university is ok’ (Male, NGO).	‘The strengths of it is that it is very basic to understand…we are not introducing anything that is new to the community...And we are talking about…locally available foods’… ‘And another strength is…the champions, like the model mothers we talk about… the smart schools... we are able to give the information out to others to who are able to spread it out’ (Female, Health Sector).
Opportunities for change	‘Such interventions I will recommend that it should be communal. The community should own it...the university should be seen in training community members...once the community owns it, it will be sustainable and then the community will also find resources to make it work and sustain it’ (Male, NGO). ‘I feel it is a wakeup call because you have done something in the community and you have seen what is going on. It is a wakeup call for my NGO and we are ready to partner with any organization in Ga mashie’ (Male, NGO). ‘Yes, they will but that one is a long-term process, you see... so it must be a joint, let me say a partnership with the traditional council. If it is a partnership and it is long term process, then it can really work - it can work’ (Male, Religious Leader).	‘We in the leadership, we have realised that eating junk food is resulting in a lot of sudden deaths…so we are considering it and see how best we can start some educational programmes’ (Male, Traditional Leader). ‘At times, the radio is the most important one, you know. If you start shouting on radio, they hear it. That is the only way to educate people’ (Female, Traditional Leader). ‘Oh, once they *[the community]* are educated on these things, I think they will support...when they [*leadership]* are empowered, they will be able to help in that direction. Once they know what they are to do, they will do that’ (Male, Assembly Officer). ‘On the job training is one of the sustainable ways, that we are looking at... the municipal nutrition officer, she is on the field, to take the opportunity to train people on the job to take up on these roles and fill up some of these responsibilities’ (Female, Health Sector).

The difference in scores between the two communities can be explained by a number of factors. In Ga Mashie, some community members held misconceptions around who efforts are for; assuming that efforts were only for the old and sick, rather than recognising the need for prevention efforts. In contrast, no misconceptions concerning efforts were identified in Ho Central. Additionally, engagement of community members was stronger within Ho Central; key community members act as community champions to engage others. Messages conveyed through the radio were also considered successful in Ho Central.

In both communities, key informants suggested that once programmes are established, community members would engage and assist with them to ensure their success, particularly if it is community led.

### Leadership

The level of readiness of the community ‘leadership’ was low in both Ga Mashie (3.50) and Ho Central (3.85), which places the communities at a “vague awareness stage”. This means that the leadership recognises the issue may be a concern but there is no immediate motivation to act (Table 2).

There was no general consensus amongst key informants about the concern/priority level that community leadership assigned to the consumption of unhealthy foods and beverages (Table 4). Others recognised that this was an issue of interest but no active efforts are made towards implementation, which implies that the level of concern is not high enough to translate into action. Another explanation for not engaging actively with this issue is that the community leadership prioritises other efforts (e.g. allocating funds for obtaining more classrooms or for celebrations), or other health issues such as anaemia and undernutrition in women of reproductive age.

**Table 4:** Supporting quotes for ‘leadership’

	Ga Mashie (Accra)	Ho Central (Ho)
Leadership level of concern	‘It is a concern to the traditional council so I will say 9 *[out of 10]* but nothing much has been done to address this issue...The chief is keen and interested in the health of the community so he is ready to support scaling up efforts to address this unhealthy eating problem identified in the community’ (Female, Traditional Leader).	‘We have realised that eating junk food is resulting in a lot of sudden deaths, diseases ...so we are concerned’ (Male, Traditional Leader). ‘Because we have not come together to discuss anything on consumption of unwholesome foods. Nothing like that, no…we don’t discuss anything concerning this topic. Never’ (Male, Assembly Officer). *‘*I have interacted with a couple of chiefs and traditional leaders, particularly those in my church and it’s always about the women being involved with alcohol and that kind of thing’ (Female, Religious Leader).
Leadership priority level	‘They know it is a challenge, they know it is a problem, they know people are consuming erm... junk food but they don’t go in with any intervention. They are aware of the problem but they do not move in to undertake anything’ (Male, NGO) ‘Yes, it is of much priority to the leadership of this community and we are planning to have people to come and support us to address this health problem. Because of that we try to get leaders to serve on the health boards to that we could get those support....it is a big priority to the traditional leaders’ (Female, Traditional Leader)	‘It is not a concern at all.. *[she laughs]* because there has never been, ermmm, any forum on which these issues have been discussed. Except the health personnel who come to talk. It has never been their priority’ (Female, Education Sector). ‘There is some apathy toward that commitment, especially when they feel it is not within the core responsibilities’ (Male, Health Sector). ‘They put other concerns too into practice. Usually, as a church, they target the salvation of the members than other things, but they added to, the members need to be healthy before salvation can also work’ (Male, Religious Leader). ‘We rather seek funds for more classrooms and to celebrate traditional festivals and to bring in more beers and drinks’ (Female, Religious Leader).
Leadership engagement	‘I will say that the leaders are not supporting the course of addressing the consumption of unhealthy foods and drinks. If you like you could go to the leaders of the community and arrange for a meeting and they will start asking for monetary gains before the program is even planned’ (Female, Commerce).	‘Even those that we are saying many, most of them *[leaders],* majority of them will not do it. Though they will say they will, they will participate in it, but they will not do the work they are supposed to do’ (Female, Commerce). ‘It seems motivation, in the form of personal reward is not forthcoming and there is some apathy toward that commitment, especially when they feel it is not within the core responsibilities, and their responsibilities, you know, normally for the public sector, it is our specific line of issues we are supposed to be addressing. So that the issue’ (Male, Health Sector). ‘that’s what I was saying that, when they are empowered, they will be able to help in that direction. Once they know what they are to do, they will do that’ (Male, Assembly Officer).
Key leaders	‘[Who are leaders that are supportive of addressing this issue in your community?]’ ‘The opinion leaders, the assembly members or assembly men and women, the chiefs’ (Female, Health Sector). ‘If you go to the chief, the chief will always welcome you and will always be interested in promoting these good practices in his community. Likewise, the assembly member and then the MP *[member of Parliament]’* (Male, Education Sector).	‘It should be the chief, queen mother and the elders…I have been saying no, no, no, because in our community, there is chieftaincy dispute. The community is divided into two... hopefully by the end of this year, we will seal that thing off, and we will be okay’ (Male, Assembly Officer).

Furthermore, key informants acknowledged that there was a lack of resources to support them in engaging in such programmes; they suggested that engagement would be increased if they received financial gains to start initiatives and support efforts to tackle the issue of consumption of unhealthy foods and drinks.

Influential leaders in both communities that could help gather and influence the community included members of the municipal assembly, traditional leaders (e.g. community chiefs, church leaders, opinion leaders, elders), health workers, youth leaders and teachers.

### Community climate

The scores for community climate were low across both communities. Ga Mashie was at the ‘vague awareness’ stage (score of 3.4), whereby community members believed that the consumption of unhealthy food and drinks in adolescent girls and women may be a concern but they showed no immediate motivation to act. Ho Central was slightly higher, ranked in the ‘pre-planning’ stage (score of 4.3), i.e. community members acknowledged that this issue was a concern in the community and that something needed to be done to address it (Table 2).

Among community members, the issue often only became a priority when individuals were diagnosed with an illness, because community members placed a low priority on prevention (Table 5). When programmes were available, community members tend to passively engage with efforts, but often require incentives to do so. Furthermore, low levels of literacy and high poverty rates act as barriers to behaviour change and some community members were concerned about the impact of efforts on their business.

**Table 5:** Supporting quotes for ‘community climate’

	Ga Mashie (Accra)	Ho Central (Ho)
Community level of concern	‘Most of the community members know that some of those foods may be harmful to their health but they still go ahead to consume them’ (Male, NGO).	‘It is not a concern to them because it’s like everybody does whatever he or she likes’ (Female, Commerce). ‘In conversations, where people complained and its mainly about the alcoholic beverages that the young girls and women have been taking. I haven’t heard anything being said about the, concern being said about the food, dietary habits and the rest’ (Female, Religious Leader).
Community priority level	‘It is not a priority to them. it only becomes a priority when like I said they are diagnosed hypertensive or diabetic, that is when what goes into them becomes a priority but till that time nobody thinks about it.’ … ‘Preventive health its... its... I don’t know people are not so much interested’ (Female, Health Sector).	‘I think generally we’ve been concerned more about the adolescent girls and women taking alcoholic beverages’ (Female, Religious Leader).
Community engagement	‘That is when they are contacted, some willingly would want to help but then most of them they will want to know what incentives come with it or what they will get from it after putting in their effort’ (Male, Education Sector).	‘Oh, I think a few people might [*oppose efforts].* I think those who will think it’s a danger to them, those who might think its pulling their businesses down’ (Female, NGO). ‘Their support [*the community],* usually is mobilising the people for you as well as they themselves participating in it’ (Male, Religious Leader).
Key community members	‘Once the opinion leaders contact the youth leaders in the community and they see the need and benefit of the intervention programs, then they will avail themselves to ensure that programs are well organised and education given to the community members to help address the consumption of unhealthy foods’ (Female, Commerce).	‘But I don’t think the consumers will have an issue with it but those who in charge of that kind of business, I think they will be offended and they will try their best to convince the people against what is good and just be taking their money’ (Female, NGO). ‘At times in churches, the women groups talk to their members on these issues’ (Female, Education Sector).

Within Ho Central, although there was a low concern towards the consumption of unhealthy foods and drinks in general, community members were concerned about the consumption of alcoholic beverages in adolescent girls and women*.* There were also greater variations in the views of key informants regarding community concern and priority levels and these differences explained the slightly higher readiness scores in Ho Central.

In both communities, influential community members and groups were highlighted that could boost the success of future efforts, e.g. youth groups, school clubs, church groups and women’s groups.

### Knowledge about the issue

Both communities were at the “pre-planning” stage for their level of knowledge about the issue (i.e. *occurrence, causes, consequences* and *misconceptions*) (4.10 for Ga Mashie and 4.38 for Ho Central) (Table 2).

Community members were aware that the consumption of unhealthy foods and drinks in the community was *occurring* (Table 6). They mentioned a shift in consumption practices from home cooked meals to ‘fast food’, from eating in the home to eating late at the market and they highlighted a marked increase in alcohol consumption. As a result, the community was perceived as facing increased risks of diet-related illnesses.

**Table 6:** Supporting quotes for ‘community knowledge of the issue’

	Ga Mashie (Accra)	Ho Central (Ho)
Occurrence within the community	‘the consumption of these unhealthy foods has been one of the major cause of death in our society. In the past, when we were eating the nkontomire [*Taro Leaves]*…when we were cooking with the Prekese *[Aridan; local spice]*…look at the age that our fore-fathers attained before they died. Today, the eating habit has changed - junk especially in our community here’ (Male, Youth Leader).	‘we have realised that eating junk food is leading, is resulting in a lot of sudden deaths, diseases that we haven’t, never experienced when we were young but the youth of today have all types of disease. You see a young person having stroke, which when we were young we never heard of, it’s the elderly people. The youth of today, a young girl is suffering from cancer, we never heard of’ (Female, Education Sector).
Misconceptions	‘At the CWC we talk to them about breastfeeding, don’t give water until the child is 6 months but…the grandmothers, the older women they tell them it’s a lie…they give them this funny, funny *[incorrect]* information’ (Female, Health Sector). ‘They may think... it is coming from an attack from an “enemy” [spiritual beliefs]- a particular example being stroke. Other people may think that it is not from my eating habit or... alcohol that is leading me to experience this but they may think that it is somebody who is using juju or black magic to curse them with such a disease’ (Male, NGO).	‘At times when you tell them, some may tell you I have been eating this for a long time and I am okay so… continuous talk and citing certain people that have done the right thing and are living healthy, will give them the idea or will alert them to desist from or to eat good’ (Female, Education Sector).
Causes	‘These unhealthy foods are bought and sold here in the community at low prices and people are buying because of the prices’ (Female, Traditional Leader). ‘There are unhealthy foods sold all over us here. Anywhere you go the foods sold there are unhealthy’ (Male, Religious Leader).	‘We see a lot of adverts, we see things that, if we were to say eat some kenkey with ‘amaa detsi’ *[green vegetable stew]* or something, you will prefer a bottle of coke and then you also fill that you are trending. So, I think the food choices’ (Female, Health Sector).
Consequences	‘They don’t know the consequences. It is only when they have the condition and even when they get the condition they don’t even know’ (Female, Health Sector). ‘They know the consequences of taking in unhealthy foods and drinks because we tell them but the sad thing is they hear but they will not do what you are telling them to do’ (Female, Health Sector).	‘I think now, knowledge is growing. I spoke to some people who told me they have been listening to one dietitian who comes, or he is on radio every, once a week to talk about these things. Talking about them’ (Female, Religious Leader).
Prevention	‘A lot do know that by mass education and public health information. Also by forming health clubs and groups and they will be the disciples of announcing the good and bad foods in the community’ (Male, Traditional Leader). ‘What can be done to prevent?... the thing is they don’t even know whether what they are taking is healthy or not. They don’t know whether they are taking is not healthy.... because they don’t know how will they know how to prevent it’ (Female, Health Sector).	‘hmmm, nothing can be done. Because if they have made their mind up. they enjoy the fat’ (Female, Traditional Leader).

Key informants disagreed about the level of community knowledge of *causes* of consumption of unhealthy foods and beverages, ranging from knowing nothing to knowing a lot. Causes discussed were: availability of unhealthy foods and drinks, advertisement of unhealthy products, cost of food, poverty, level of education, peer influences and social and environmental influences.

Key informants also disagreed on the level of community knowledge of the *consequences* of consuming unhealthy foods and beverages, ranging from knowing nothing to knowing a lot. Some community members may know that unhealthy food and drink consumption impacts upon health but may not know the specific consequences, although those who are educated may have a greater understanding. Some community members only learn about the consequences once they are affected with diet-related diseases. However, key informants mentioned that knowledge of the consequences may be growing due to information being provided in the media and via health workers.

Key informants described *misconceptions* that existed within both communities around the consumption of unhealthy foods and drinks. These included whether specific food and drink items were harmful (e.g. all foreign imported foods were viewed as healthy); to whom the items were harmful (e.g. items that harm only the old, items that should or should not be consumed during pregnancy/lactation); consequences of unhealthy food and drink consumption; causes of illness (e.g. spiritual or supernatural powers) and social connotations associated with particular food items (e.g. wealth associated with consumption of certain foods/beverages). It is believed that misconceptions originated from older family members or from people who have consumed unhealthy products for a long period of time, but have not developed any illness.

### Resources

Both communities were at the ‘vague awareness stage’ for resources, (3.08 for Ga Mashie and 3.35 for Ho Central), which means that overall there were limited resources identified that could be leveraged for further efforts to tackle the issue (Table 2). Some resources were identified as clear barriers to leaders engaging with or implementing actions to address the issue (i.e. money, time, and experts or volunteers in the community) (Table 7). In terms of funding, informants reported that there were zero to limited funds available (e.g. money from individual donors, community contributions and local levies) and the funding from ministries was deemed inconsistent. The community key informants also mentioned that they would expect subsistence allowances to support various programmes but this was not currently in place and therefore hindered leadership engagement. Key informants reported that businesses and organisations in the area would not financially support such efforts and that donor support is overall reducing.

**Table 7:** Supporting quotes for ‘resources’

	Ga Mashie (Accra)	Ho Central (Ho)
Action or inaction to mobilise resources	‘If we are saying community leadership purposively going around to mobilize these volunteers for future programmes...They are not going to them in frantic efforts to get money to upscale or improve upon certain interventions that are ongoing in the community’ (Male, NGO).	‘Once they know that these resources are available, the experts, the will make use of them to support’ (Male, NGO). ‘because at times when you put the effort there they don’t get so they feel reluctant to do’ (Male, Religious Leader).
Information	‘I have quite a number of brochures and all that about anaemia in pregnancy, what to eat, what not to eat but I have never sent out to the community to give out... It is only at the facility ‘Female, Health Sector	‘Normally they are graphic representations, and they are normally at institutions like the hospitals’... ‘But within community you are hardly to get such’ (Male, Health Sector). ‘I think it’s just verbal because I don’t know of any newspaper or article or anything. Posters that...It’s just verbal I know and radio’ (Female, NGO).
Money	‘I must be honest with you, anything that has to do with funding just take it off because they will go in for something for themselves not for you. You see, you are coming in for a programme then they expect that you have your luggage full of cedis to support them rather’ (Male, Religious Leader).	‘for funding…it’s the community, from our own meagre resources…We usually have contributions from the community, we have our own levies’…‘so if we need any money, that is where we will go to’ (Male, Traditional Leader). ‘Now that we say we are a middle-income country, that is what we are touting ourselves as a country so yeah, donor support is reducing drastically’ (Female, Health Sector).
Organisations	‘We have some NGOs around and…they will support in addressing the consumption of unhealthy foods and drinks.’ (Male, Traditional Leader) ‘Yes, we have community groups, we have women groups, we have hairdressers’ association, we have church women groups…we will meet with all of them…they will give us time’ (Female, Health Sector).	‘I know Pencils of Promise, but we deal with only school, I know PLAN Ghana is also there, they also do health education and which other NGO? I don’t know some of the NGOs. I wouldn’t say a lot. I will say few or there’s no few there?’ (Female, NGO).
Experts	‘Ok so we have people that academically they have the expertise and then we…the food and drugs board…they…have the technical experts’ (Male, NGO). ‘We have some people here who are experts in the community that could help like the health officials’ (Female, Traditional Leader).	‘As for the experts, we have nurses, both community and public health nurses, we have food and nutrition, ermm, teachers and nutritionists. So, they are available, so some’ (Female, Education Sector). ‘so, we have a lot of them (experts) who are there. But just that maybe those who will be willing to do it will be the problem’ (Female, NGO).
Volunteers	‘I will say that once the health programs are started…the volunteers will be available to support the program to be a success and also help to sustain those efforts’ (Female, Traditional Leader). ‘Like I said earlier, if you motivate the volunteers with some incentives, they will avail themselves to help’ (Male, Religious Leader).	‘Volunteers, will I say some?... I say some because, there are a lot of people who, even I do it myself, can you come to this place and talk about this, and I go like oh I don’t have the time or something. So, people are not willing, they complain about time, they complain about not being able to’ (Female, NGO).
Space	‘A lot of spaces are available here in the community. We have churches, parks like the Mantse Agboona Park, London market area, lots of places here to be used for the health education activities’ (Male, Traditional Leader).	‘space, maybe, we have the radio stations, the media that could be used address this issue, we have the churches and the schools, and the traditional rulers place and they can create the avenues for such news’ (Female, Religious Leader).
Time	‘if we could have... more time... maybe you have 30 min so sometimes the mothers don’t have time to ask questions’ (Female, Health Sector). ‘The leaders will rather be talking about using their time for other profitable ventures’ (Female, Traditional Leader).	‘So, if you’re sitting down listening to education, you feel that you are wasting your time…why won’t I use that time to go and prepare something for sale…even those who are doing nothing, they don’t also have time...the radios, people hear everything, they listen’ (Female, Traditional Leader).

Several key informants discussed time as a barrier to engaging in programmes. For example, health visits were deemed too short to allow women to ask questions. Community leaders reported that they may prioritise their time for other matters. When volunteers are recruited to help, they often refuse on the basis of not having enough time. It is also believed that some community members may not make time to attend programmes around healthy eating as they would rather spend their time working or looking for work. A few key informants mentioned that radio messages were perceived as more successful because community members could listen whilst doing other activities.

Other resources such as information, organisations, space and experts/volunteers currently exist and could be further leveraged to address the issue of consumption of unhealthy foods and beverages in the communities. Some key informants mentioned that there may be some posters addressing the consumption of unhealthy foods and beverages displayed in the communities but only in certain places (e.g. health care facilities and schools). Additional information on the topic was shared via radio programmes but reach may be limited as not all community members own a radio. Information vans are also a way of sharing information within the community. Nurses and health workers have booklets for mothers and carers but it is unclear if these address the issue of consumption of unhealthy foods and drinks specifically.

Key informants also identified organisations (e.g. Non-Governmental Organisations (NGOs), youth groups, women’s group, food vendors’ association and other community groups), currently not mobilised to address the consumption of unhealthy foods and drinks in the communities, that could help support such efforts. Some of these organisations may already be involved in one-off efforts, but there was no sustainable involvement due to a lack of financial resources.

There were a number of experts identified in the community that could be engaged to support programmes around healthy eating and drinking, e.g. public health and community nurses trained in basic nutrition, dietitians, health promotion officers, School Health Education Programme coordinators, Food and Drug Administration workers, nutrition officers, sanitary inspectors, NGOs and University researchers. However, not all experts will be willing to support such efforts. Space is not a limiting factor, as a lot of space was available in both communities to implement efforts to address the consumption of unhealthy foods and drinks. Churches, parks, municipal buildings, stadium, community centres and schools could be utilised for a given intervention.

## Discussion

This study investigated how ready urban communities are to improve the diets of women of reproductive age in Ghana. The specific objectives were: (i) to determine the stage of readiness of community key informants to address the consumption of unhealthy foods and beverages in adolescent girls and women of reproductive age in two deprived communities at different stages of the nutrition transition in urban Ghana, and (ii) to gain qualitative insights of community factors that could affect the implementation of interventions targeting unhealthy food and beverage consumption.

Whilst consumption of unhealthy foods and beverages was defined as the consumption of energy-dense, nutrient-poor products, key informants referred to unhealthy diets in terms of both nutritional balance and food safety. Actions discussed in the interviews therefore referred to unhealthy in a holistic sense and demonstrate that food hygiene is a concern in the target communities. Despite their different stages of nutrition transition, the mean community readiness scores were similar between the two communities. These indicated that both communities were at a “vague awareness stage”, highlighting that they are not yet ready to address the issue of unhealthy food and beverage consumption in women of reproductive age. This level of readiness reflects the fact that “most key informants feel there is a local concern about the issue under investigation, but there is no immediate motivation to do anything about it” [11]. This result also suggests that a higher prevalence of overweight/obesity in Ga Mashie, compared to Ho Central, has not yet translated into accelerated readiness for intervention.

Within each community, the scores observed across dimensions were quite similar, indicating a relatively low level of readiness for all dimensions of the model. Plested et al. [11] state that for an intervention to be effective, each dimension should be at an equal stage of readiness. Therefore, based on our findings, an initial focus should be on increasing the community knowledge of efforts (Ga Mashie) and the resources available (Ho Central).

Our study is the first to assess the stage of community readiness to address unhealthy diets in Sub-Saharan Africa or other LMICs, making relevant comparisons difficult. However, studies utilising the CRM for obesity prevention interventions have been conducted in South Africa [25] and in several HICs [15, 16, 18, 19, 32, 33], with some studies focusing specifically on deprived communities [20, 21, 34]. The overall readiness stage (i.e. “vague awareness”) found in the current study is higher than the one reported in South Africa (i.e. “denial/resistance stage”) [25]. In both studies, community knowledge of efforts was one of the lowest scoring dimensions. In the South African study, the highest scores were seen for resources (3.75), followed by knowledge of the issue (3.18) [25] whereas in the present study, the highest readiness scores were seen for knowledge of the issue (4.10) and leadership (3.50) (Ga Mashie) and for knowledge of the issue (4.38) and community climate (4.29) (Ho Central).

Findings from our study were also comparable to those found in deprived communities in HICs [20, 21, 34]. As in the current study, the readiness score for the prevention of adolescent obesity in a Latino community in Nebraska was 3, corresponding to the “vague awareness” stage [34]. However, in contrast to the present study, the highest scores were observed for community knowledge of efforts and resources [34], which were the lowest dimensions in our study. In comparison to studies conducted in non-deprived communities in HICs, the stage of community readiness in our study was either similar [16, 18] or consistently lower [15, 19]. For example, a UK study aimed at tackling obesity amongst pre-adolescent girls found much higher readiness scores for implementing both physical activity (6.08; “initiation stage”) and dietary interventions (5.74; “preparation stage”) [19].

### Strengths and limitations

This study is novel as it is one of the first studies to assess community readiness in the African context prior to designing and implementing interventions to reduce consumption of unhealthy foods and beverages. A further strength of this study is the thematic qualitative analysis that complemented the quantitative scoring, which provided crucial information that can be leveraged to design and implement effective interventions on healthy eating in the two target communities, which showed a similar level of community readiness.

This study also has some limitations. Including views from only two deprived communities (Ga Mashie and Ho Central) may not be representative of the whole of Accra and Ho. Scoring CRM interviews using pre-defined anchored rating statements has been criticised [35, 36] for permitting too much researcher subjectivity. In order to reduce this potential bias and ensure inter-rater reliability, the CRM interviews were scored independently by two reviewers and then combined. If discrepancies arose between reviewers, these were discussed until consensus was reached. By only focusing on key informants in the community, the CRM places responsibility for health-related issues on the shoulders of communities, and therefore does not engage with other key decision-makers in government.

### Implications for future interventions

This study has identified avenues for potential implementation of future interventions. Our findings suggest that community members are relatively knowledgeable about this issue. It was acknowledged that there are some unhealthy food practices occurring within the communities, including increased consumption of ‘fast foods’, eating out, and excessive alcohol consumption. However, there was less knowledge about the specific health consequences of these behaviours. There was recognition from the communities that the consumption of unhealthy foods and beverages is an issue that needs to be tackled (particularly in Ho Central), but this concern does not necessarily translate into prioritisation and action. Key informants interviewed were also concerned that any programmes focusing on improving food hygiene practices and reducing the consumption of unhealthy food and beverages would impact negatively on local businesses and consequently, on community members’ livelihoods. This suggests a need to increase awareness of the issue to ensure it is prioritised and resources are mobilised sensitively to address the issue, whilst including local businesses to ensure that they can continue to trade using healthier food options. This would minimise negative economic consequences of any intervention on the communities concerned. Additionally, it may be necessary to target community members’ understanding of non-communicable disease prevention, emphasising that prevention strategies are preferable to treatment, and a worthwhile investment.

The low scores for community knowledge of efforts implies that there is a need to develop initiatives further, ensuring that these are scaled-up and actively shared within the communities. Any actions that do exist to reduce unhealthy eating are currently targeted at specific population groups and there is a need for interventions that support healthy eating specifically in women of reproductive age.

The relatively low scores for resources suggest that they may act as barriers to implementing interventions. However, it is important to note that there is potential for some existing resources to be leveraged further to support efforts, e.g. information, expert/volunteers, organisations and space. There is a need to consider low-cost strategies and potential support from existing NGOs to administer programmes. Coordinating existing experts and NGO activity and utilising widely available community spaces for information sharing would maximise efforts. Other potential people who might be mobilised to support interventions are members of the municipal assembly, health workers, youth leaders, teachers and traditional leaders, e.g. community chiefs, church leaders, opinion leaders, elders and women’s groups. Information sharing could take place through the radio, as this is seen as a potentially positive delivery route because people can listen to it whilst working. Information contained in booklets or posters, currently displayed in health facilities and schools, could be placed more strategically to ensure a broader community outreach than is currently being achieved.

## Conclusions

Despite some awareness that the consumption of unhealthy foods and beverages is an issue amongst women of reproductive age in urban Ghana, tackling unhealthy diets is not yet a priority for intervention, except on a small scale to specific target groups. For both communities, the community readiness assessment suggests that there is a need to raise further awareness regarding the issue prior to implementing initiatives to improve diets. Key stakeholders and groups have been identified, who could be engaged to develop low-cost strategies for raising awareness through channels suggested by the communities. Examples of such channels include community nurses, radios, information buses and posters. The focus should be on raising the awareness that non-communicable diseases are preventable with lifestyle changes and that making these changes will benefit the whole community. Simultaneously, efforts should be made to tackle the issue of food hygiene, which was recognised as both a high concern and priority. If not addressed, this could be a potentially limiting factor in making healthy food choices, e.g. sliced fruits and vegetables that are promoted as part of a healthy diet can become unhygienic and potentially unhealthy if washed in unclean water. This last factor emphasises a unique additional challenge in designing interventions to reduce the rise of diet-related non-communicable diseases in communities undergoing nutrition transition in LMICs. There is a need to ensure such communities are ready for interventions to improve their diets prior to implementation. These communities need to be serviced with interventions that are sensitive to, and include members of the local trading environment and that also incorporate food safety and hygiene, if they are to be successful at reducing diet- related non-communicable diseases.

## Additional files

Additional file 1: CRM Interview Guide. (DOCX 36 kb)

Additional file 2: CRM Codebook for analysis. (DOCX 23 kb)

## Abbreviations

CRM: Community Readiness Model
HICs: High income countries
LMICs: Low- and middle-income countries
NGOs: Non-Governmental Organisations
