# Supplementary material for: How ready are communities to implement actions to improve diets of adolescent girls and women in urban Ghana?
Source: BMC Public Health. 2019 May 28;19:646. doi: 10.1186/s12889-019-6989-5 (PMC6537223; doi:10.1186/s12889-019-6989-5)
Supplement: Supplementary file 1 — CRM Interview Guide. (DOCX 36 kb) [file 12889_2019_6989_MOESM1_ESM.docx]

Additional file 1: CRM Interview Guide

**Introduction**

Hello, my name is __________ from (organisation name).

Thank you so much for agreeing to be interviewed for this project. We are contacting key people to ask about *the consumption of unhealthy foods and beverages* as it occurs in *the Ho central areal/Ga Mashie area*. The entire process, including individual names, will be kept confidential.

Just to be clear, when I refer to consumption of unhealthy foods and beverages, I specifically mean:

consumption of products such as processed meats; sugar and sweet spreads; cakes and sweets; sodas and sweetened beverages; fried potatoes/yam/plantain; oils; spreading fats; cooking fats; condiments.

In addition, I would like you to answer specifically about the community of Ho central/Ga Mashie area (add details here)*.*

I would like to record our interview, so that we can get an accurate representation of what you’ve said. Would that be okay with you?

**Community Readiness Interview Questions**

In many questions in this survey I am going to ask you to respond to questions in relation to a scale. The scale will be from 1 to 10 and you will need to respond according to how much you agree with that particular question.

For example, a question might ask: How much do you like Red Red? You will need to respond on the scale from 1 to 10. If you very much like Red Red, then you would respond “10”. But if you don’t like Red Red at all you would respond with a “1”. If you like it a little bit then you would respond with a “3” or if you somewhat like it then you would respond with “5” or “6” and if you like it you would respond “7” or “8”.

**1. For the following question, please answer keeping in mind your perspective of what community members believe and not what you personally believe.**

**On a scale from 1-10, how much of a concern is** the consumption of unhealthy foods and beverages **to members of** *Ho Central/Ga Mashie community****,*** **with 1 being “not a concern at all” and 10 being “a very great concern”?** *(Scorer note: Community Climate)*

1 🞎 2 🞎 3 🞎 4 🞎 5 🞎 6 🞎 7 🞎 8 🞎 9 🞎 10 🞎

Not at all Somewhat Very much

**Can you tell me why you think it’s at that level?**

*Interviewer: Please ensure that the respondent answers this question in regards to* ***community members*** *not in regards to themselves or what they think it should be.*

***COMMUNITY KNOWLEDGE OF EFFORTS***

I’m going to ask you about current community efforts to address *consumption of unhealthy foods and beverages*. By efforts, I mean any programmes, activities, or services in your community that address *consumption of unhealthy foods and beverages*.

**2. Are there efforts in** (Ho central/Ga Mashie *community)* **that address** the consumption of unhealthy foods and beverages**?**

*If* ***Yes****, continue to question 3; if* ***No or Don’t Know****, skip to question 16.*

**3. Can you briefly describe each of these?**

*Interviewer: Write down names of efforts so that you can refer to them in #4-5 below.*

4. How long have each of these efforts been going on? *Probe for each program/activity.*

5. Who do each of these efforts serve (e.g., a certain age group, ethnicity, etc.)?

**6. About how many community members are aware of each of the following aspects of the efforts**

**- none, a few, some, many, or most?**

- **Have heard of efforts?**

**□ None □ a few □ some □ many □ most □ don’t know**

- **Can name efforts?**

**□ None □ a few □ some □ many □ most □ don’t know**

- **Know the purpose of the efforts?**

**□ None □ a few □ some □ many □ most □ don’t know**

- **Know who the efforts are for?**

**□ None □ a few □ some □ many □ most □ don’t know**

- **Know how the efforts work (e.g. activities or how they’re implemented)?**

**□ None □ a few □ some □ many □ most □ don’t know**

- **Know the effectiveness of the efforts?**

**□ None □ a few □ some □ many □ most □ don’t know**

**7. Thinking back to your answers, why do you think members of your community have this amount of knowledge?**

**8. Are there misconceptions or incorrect information among community members about the current efforts?** *If yes:* What are these?

9. How do community members learn about the current efforts?

10. Do community members view current efforts as successful?

*Probe:* What do community members like about these programmes? What don’t they like?

11. What are the obstacles to individuals engaging with these efforts?

12. What are the strengths of these efforts?

13. What are the weaknesses of these efforts?

15. What planning for additional efforts to address the consumption of unhealthy foods and beverages is going on in Ho central/Ga Mashie community?

***Only ask #16 if the respondent answered “No” to #2 or was unsure****.*

**16. Is anyone in** *Ho central/Ga Mashie community* **trying to get something started to address** the consumption of unhealthy foods and beverages? **Can you tell me about that?**

***LEADERSHIP***

I’m going to ask you how the leadership in *Ho central/Ga Mashie* *community* perceives *the consumption of unhealthy foods and beverages*. By leadership, we are referring to people who have power to make decisions about this issue or have ability to influence actions or lead the community to address the issue.

**17. Using a scale from 1-10, how much of a concern is** the consumption of unhealthy foods and beverages **to the leadership of** *Ho central/Ga* Mashie *community***, with 1 being “not a concern at all” and 10 being “a very great concern”?**

1 🞎 2 🞎 3 🞎 4 🞎 5 🞎 6 🞎 7 🞎 8 🞎 9 🞎 10 🞎

Not at all Somewhat Very much

**Can you tell me why you say it’s (***not at all of a concern/somewhat of a concern/very much of a concern***)?**

**17a. How much of a priority is addressing** the consumption of unhealthy foods and beverages **to the leadership?**

1 🞎 2 🞎 3 🞎 4 🞎 5 🞎 6 🞎 7 🞎 8 🞎 9 🞎 10 🞎

Not at all Somewhat Very much

**Can you explain why you say this?**

**18. I’m going to read a list of ways that leadership might show its support or lack of support for efforts to address** the consumption of unhealthy foods and beverages**.**

**Can you please tell me whether none, a few, some, many or most leaders would or do show support in this way? Also, feel free to explain your responses as we move through the list.**

**How many leaders…**

- **At least support efforts without necessarily being active in that support?**

**□ None □ a few □ some □ many □ most**

**- Participate in developing, improving or implementing efforts? (For example, by being a member of a group that is working toward these efforts.)**

**□ None □ a few □ some □ many □ most**

- **Support allocating resources to fund community efforts?**

**□ None □ a few □ some □ many □ most**

- **Play a key role as a leader or driving force in planning, developing or implementing efforts?**

**□ None □ a few □ some □ many □ most**

**(prompt: How do they do that?)**

- **Play a key role in ensuring the sustainability of community efforts? (For example by allocating long-term funding.)**

**□ None □ a few □ some □ many □ most**

**19. Does the leadership support scaling up efforts in the community to address** the consumption of unhealthy foods and beverages**?**

*If* ***yes****:* **How do they show this support? (For example, by supporting, by being involved in developing the efforts, or by being a driving force or key player in achieving these scaling up efforts?)**

20. Who are leaders that are supportive of addressing this issue in your community?

21. Are there leaders who might oppose addressing the consumption of unhealthy foods and beverages? How do they show their opposition?

***COMMUNITY CLIMATE***

For the following questions, again please answer keeping in mind your perspective of what community members believe and not what you personally believe.

**22. How much of a priority is addressing this issue to community members?**

1 🞎 2 🞎 3 🞎 4 🞎 5 🞎 6 🞎 7 🞎 8 🞎 9 🞎 10 🞎

Not at all Somewhat Very much

**Can you explain your answer?**

**23. I’m going to read a list of ways that community members might show their support or their lack of support for community efforts to address** the consumption of unhealthy foods and beverages**.**

**Can you please tell me whether none, a few, some, many or most community members would or do show their support in this way? Also, feel free to explain your responses as we move through the list.**

**How many community members…**

- **At least support community efforts without being active in that support?**

**□ None □ a few □ some □ many □ most**

- **Participate in developing, improving or implementing efforts? (For example by attending group meetings that are working toward these efforts.)**

**□ None □ a few □ some □ many □ most**

- **Play a key role as a leader or driving force in planning, developing or implementing efforts?**

**□ None □ a few □ some □ many □ most**

**(prompt: How do they do that?)**

**How many community members…**

- **Are willing to pay more (for example, in taxes) to help fund community efforts?**

**□ None □ a few □ some □ many □ most**

**24. About how many community members would support scaling up efforts in the community to address** the consumption of unhealthy foods and beverages**? Would you say none, a few, some, many or most?**

**□ None □ a few □ some □ many □ most**

*If more than none:* **How might they show this support? (For example, by passively supporting or by being actively involved in scaling up the efforts?)**

25. Are there community members who oppose or might oppose addressing the consumption of unhealthy foods and beverages? How do or will they show their opposition?

***KNOWLEDGE ABOUT THE ISSUE***

28. On a scale of 1 to 10 where a 1 is no knowledge and a 10 is detailed knowledge, how much do community members know about consumption of unhealthy foods and beverages?

1 🞎 2 🞎 3 🞎 4 🞎 5 🞎 6 🞎 7 🞎 8 🞎 9 🞎 10 🞎

None Somewhat A lot

Why do you say it’s a ____?

**29. Would you say that community members know nothing, a little, some or a lot about each of the following as they pertain to** the consumption of unhealthy foods and beverages? (*After each item, have them answer.)*

**29a. How much would you say that community members know about consumption of unhealthy foods and beverages, in general (prompt as needed with “nothing, a little, some or a lot”).**

**□ Nothing □ a little □ some □ a lot**

**29b. How much would you say that community members know about the causes of consuming unhealthy foods and beverages?**

**□ Nothing □ a little □ some □ a lot**

**29c. How much would you say that community members know about the consequences of consuming unhealthy foods and beverages?**

**□ Nothing □ a little □ some □ a lot**

**29d.** **How much would you say that community members know about whether people do consume unhealthy foods and beverages?**

**□ Nothing □ a little □ some □ a lot**

**29e. How much would you say that community members know about what can be done to prevent consumption of unhealthy foods and beverages**

**□ Nothing □ a little □ some □ a lot**

**30. What are the misconceptions among community members about** the consumption of unhealthy foods and beverages?

31. What type of information is available in Ho central/Ga Mashie community about the consumption of unhealthy foods and beverages (e.g. newspaper articles, brochures, posters)?

*If they list information, ask:* Do community members access and/or use this information?

***RESOURCES FOR EFFORTS*** *(time, money, people, space, etc.)*

*If there are efforts to address the issue locally, begin with question 32. If there are no efforts, go to question 33.*

**32. How are current efforts funded? Is this funding likely to continue into the future?**

**33. I’m now going to read you a list of resources that could be used to address** the consumption of unhealthy foods and beverages consumption of unhealthy foods and beverages **in your community. For each of these, please indicate whether there is none, a little, some or a lot of that resource available in your community that could be used to address** the consumption of unhealthy foods and beverages**?**

- **Volunteers?**

**□ None □ a little □ some □ a lot □ don’t know**

- **Money from organisations and/or businesses?**

**□ None □ a little □ some □ a lot □ don’t know**

- **Government funding?**

**□ None □ a little □ some □ a lot □ don’t know**

- **Experts?**

**□ None □ a little □ some □ a lot □ don’t know**

- **Space?**

**□ None □ a little □ some □ a lot □ don’t know**

**- NGO**

**□ None □ a little □ some □ a lot □ don’t know**

**34. Would community members and leadership support the use of these resources to address** the consumption of unhealthy foods and beverages?  **Please explain.**

**35. On a scale of 1 to 5, where 1 is no effort and 5 is a great effort, how much effort are community members and/or leadership putting into each of the following areas to increase the resources going toward addressing** the consumption of unhealthy foods and beverages **in your community?**

**- On a scale of 1 to 5, where 1 is no effort and 5 is great effort, how much efforts are community members and/or leadership putting into seeking volunteers for current or future efforts to address consumption of unhealthy foods and beverages in the community?**

**- On a scale of 1 to 5, where 1 is no effort and 5 is great effort, how much efforts are community members and/or leadership putting into soliciting donations from businesses or other organizations to fund current or scaling up community efforts?**

**- On a scale of 1 to 5, where 1 is no effort and 5 is great effort, how much efforts are community members and/or leaderhip putting into applying for funding from government to address the consumption of unhealthy foods and beverages** **in the community?**

**- On a scale of 1 to 5, where 1 is no effort and 5 is great effort, how much efforts are community members and/or leadership putting into training community members to become experts?**

**- On a scale of 1 to 5, where 1 is no effort and 5 is great effort, how much efforts are community members and/or leadership putting into recruiting experts to the community?**

**36. Are you aware of any action plans that have been submitted for funding to address** the consumption of unhealthy foods and beverages **in** Ho central/Ga Mashie *community***?**

***If Yes:* Please explain.**

**Demographics of respondent (optional)**

1. Gender:

2. What is your work title? _______________________________________

3. What is your age range? -

___ < = 18

___ 19-24 ___ 25-34

___ 35-44 ___ 45-54

___ 55-64 ___ 65 and above

4. Do you live in Ho central/Ga Mashie community?

□ Yes □ No

If no: What community? ________________

5. How long have you lived in your community? ________________________

6. Do you work in (community)?

□ Yes □ No

If no: What community? ________________

7. Do you live in (community)?

□ Yes □ No

If no: What community? ________________
