# Supplementary material for: How ready are communities to implement actions to improve diets of adolescent girls and women in urban Ghana?
Source: BMC Public Health. 2019 May 28;19:646. doi: 10.1186/s12889-019-6989-5 (PMC6537223; doi:10.1186/s12889-019-6989-5)
Supplement: Supplementary file 2 — CRM Codebook for analysis. (DOCX 23 kb) [file 12889_2019_6989_MOESM2_ESM.docx]

Additional file 2: CRM Codebook for analysis

| Name | Description |
| --- | --- |
| **Community Climate** |  |
| Community Engagement | Community engagement with the efforts - supporting, volunteering, key driving forces for efforts, passive efforts etc |
| Community Level of Concern |  |
| Community Priority Level | Priority defined as knowing it's a concern and addressing this over other issues or choosing to tackle other issues over healthy eating |
| Key Community Members |  |
| **Community Knowledge of Efforts** |  |
| Programmes |  |
| Community Engagement & Reach  Target group  Type and content |  |
| Duration and time |  |
| Facilitator |  |
| Misconceptions about the efforts |  |
| Obstacles | Obstacles to individuals engaging with the efforts e.g. personal money, space, resources, resistance to engage with behaviours |
| Opportunities | Facilitators and positive elements already in the community that would aid with programmes (opposite of obstacles) |
| Strengths |  |
| Weaknesses |  |
| **Knowledge about the Issue** |  |
| Causes |  |
| Consequences |  |
| Misconceptions |  |
| Occurrence within the community | Knowledge of level of occurrence of issue within the community |
| Prevention | Awareness of appropriate measures to prevent unhealthy diet |
| **Leadership** |  |
| Key Leaders |  |
| Leadership engagement |  |
| Leadership level of concern |  |
| Leadership Priority Level |  |
| **Resources for Efforts** |  |
| Action or inaction to mobilise resources |  |
| Information | Tangible information resources e.g. radio, posters, flyers |
| Money |  |
| Organisations |  |
| People |  |
| Experts |  |
| Volunteers |  |
| Space |  |
| Time |  |
